# No Interface, No Problem: Gesture Recognition on Physical Objects Using Radar Sensing

**DOI:** 10.3390/s21175771

**Published:** 2021-08-27

**Authors:** Nuwan T. Attygalle, Luis A. Leiva, Matjaž Kljun, Christian Sandor, Alexander Plopski, Hirokazu Kato, Klen Čopič Pucihar

**Affiliations:** 1 Faculty of Mathematics, Natural Sciences and Information Technologies (FAMNIT), University of Primorska, Glagoljaška 8, 6000 Koper, Slovenia; nuwan.attygalle@famnit.upr.si (N.T.A.); matjaz.kljun@upr.si (M.K.); 2 Department of Computer Science, University of Luxembourg, Maison du Nombre 6, Avenue de la Fonte, L-4364 Esch-sur-Alzette, Luxembourg; name.surname@uni.lu; 3 School of Creative Media, City University of Hong Kong, Hong Kong, China; csandor@cityu.edu.hk; 4 Department of Information Science, University of Otago, P.O. Box 56, Dunedin 9054, New Zealand; alexander.plopski@otago.ac.nz; 5 Graduate School of Science and Technology, Nara Institute of Science and Technology, Takayama 8916-5, Ikoma, Nara, Japan; kato@is.naist.jp

**Keywords:** radar sensing, gesture recognition, deep learning, human factors

## Abstract

Physical objects are usually not designed with interaction capabilities to control digital content. Nevertheless, they provide an untapped source for interactions since every object could be used to control our digital lives. We call this *the missing interface* problem: Instead of embedding computational capacity into objects, we can simply detect users’ gestures on them. However, gesture detection on such unmodified objects has to date been limited in the spatial resolution and detection fidelity. To address this gap, we conducted research on micro-gesture detection on physical objects based on Google Soli’s radar sensor. We introduced two novel deep learning architectures to process range Doppler images, namely a three-dimensional convolutional neural network (Conv3D) and a spectrogram-based ConvNet. The results show that our architectures enable robust on-object gesture detection, achieving an accuracy of approximately 94% for a five-gesture set, surpassing previous state-of-the-art performance results by up to 39%. We also showed that the decibel (dB) Doppler range setting has a significant effect on system performance, as accuracy can vary up to 20% across the dB range. As a result, we provide guidelines on how to best calibrate the radar sensor.

## 1. Introduction

**Figure 1:**
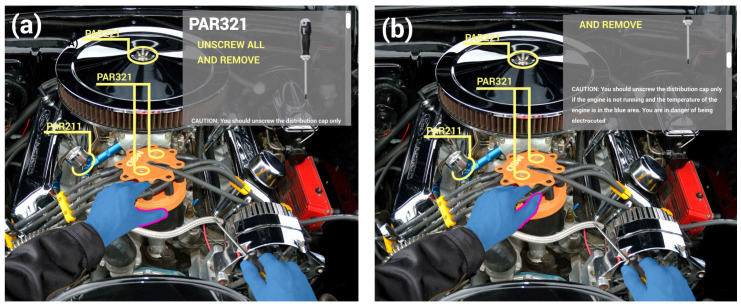
Application scenario illustrating our envisioned interactions. A mechanic who is following AR instructions visible though a head mounted display (**a**) can swipe on the nearby cable to (**b**) scroll down the instructions view.

The vast majority of physical objects are not designed with interaction capabilities in mind [1]. Nevertheless, all these objects could be used to interact with digital content, and thus provide an untapped source for interaction. A current approach is to add computational capabilities to objects to make them “smart” and to enable us to control some aspects of our digital life. However, if we could detect gestures on arbitrary objects, it would dramatically increase the input options for users. For example, imagine a maintenance task where instruction annotations are directly projected onto the object in need of repair, such as in Figure 1. We could execute different gestures on the object to perform a variety of tasks, including to browse the maintenance instructions, query additional information, or provide feedback in order to communicate a problem to a remote expert.

If objects are not instrumented in any way, on-object gesture detection becomes difficult. Widely used sensing systems that require a direct line of sight (such as vision-based sensing systems using RGB or RGB-D cameras) can only recognise gestures executed on the visible part of the object, limiting the range of possible interactions. For example, if the user holds the object in their hand, only the visible thumb can be used for interaction.

Radar-based sensing has the promise of enabling spontaneous interaction with surfaces even when the hand is occluded by the object. It does not require a direct line of sight since electromagnetic waves used in radar sensing can propagate through non-conductive materials. However, gesture detection on objects using radar signals also introduces several challenges.

First of all, the object one interacts with will introduce noise and distortions into the radar signal. The level of signal degradation will depend on the properties of materials that the object is made of, mainly the signal attenuation and transmission coefficient [2]. The higher the attenuation, the more opaque the object is to the radar sensor, which reduces the sensor’s ability to acquire meaningful information about gestures. Research exploring how signal degradation affects on-object gesture detection performance with the radar sensor is nearly non-existent. To the best of our knowledge, an exception is that of our previous work [1] where we showed that gesture detection on objects is not practical with standard machine learning approaches (random forest and support-vector machine classifiers) and core features provided by the Soli SDK.

In addition, we need to consider the sensitivity of the radar used for gesture detection. When measuring a radar signal, the amount of energy sent and received are compared by the sensor antennas. The decibel (dB) range that will be considered for gesture detection, can thus have a significant impact on the system’s ability to detect and distinguish different gestures. For example, a small dB range will remove noise from the signal, but also reduce the sensitivity of the sensor since the sensor will only see reflections in which little energy is lost, thus showing only “large objects” (i.e., objects that reflect a lot of energy). This may result in the loss of important information, especially in situations where materials occlude the hand and fingers executing gestures. Sensor calibration is therefore difficult but also very important for optimal system performance.

These challenges open up several research questions we address in this paper: Can radar sensing be used for robust on-object gesture detection? How does the dB range affect gesture detection performance and is this user-dependent? What is the potential advantage of correctly calibrating the dB range and how does one do it? To answer these questions, we designed, implemented, and evaluated a gesture recognition system based on Google Soli’s millimetre-wave (mm-wave) radar sensing technology. We show that our system can detect different gestures on and behind objects, revealing its potential for spontaneous interactions with everyday objects. The contributions of this paper are:

- A publicly available data set of 8.8 k labelled gestures executed on a standing wooden photo frame using a wrist-mounted millimetre-wave (mm-wave) Google Soli radar sensor (see Data availability statement).
- Design and implementation of two novel model architectures that (i) achieve robust on-object gesture detection (accuracy of up to 94% on a five-gesture set); and (ii) outperform current state-of-the-art models used for radar-based mid-air gesture recognition (up to 8% improvement in mid-air gesture detection accuracy).
- A comprehensive set of experiments involving the training and evaluation of 282 classifiers showing that (i) there is a significant effect of dB range on gesture detection performance (the accuracy varies up to 20%); (ii) the effect of the dB range is user independent; and (iii) how to find an optimal dB range value.

## 2. Related Work

In this section, we discuss prior approaches to detecting mid-air and on-object gestures, with a focus on radio-frequency (RF) and millimetre-wave (mm-wave) radar sensing.

### 2.1. Gesture Detection

Two different approaches to gesture interaction are commonly used: (i) *mid-air* interaction, where interaction is separated from the object we interact with; and (ii) *on-object* interaction, where interaction is executed on the object itself.

Methods for mid-air gesture detection have been extensively explored in the past. A recent literature review [3] found 71 different methods for the classification of hand gestures where signal acquisition methods ranged from: vision-based approaches such as RBG and RGB-D [4,5]; a data glove system equipped with flex sensors, inertial sensors, and gyroscopes [6]; surface electromyography (sEMG) systems sensing muscular activity [7]; wearable wristband and rings [8]; and systems that rely on radar sensing using various frequency bands [9,10].

On-object gesture detection is more challenging, since either objects or users need to be instrumented with specific sensors in order to detect gestures. Moreover, objects add noise to the gesture detection pipeline and increase the difficulty of hand segmentation. Previous research explored several methods for on-object gesture detection, such as infrared proximity sensors allowing, for example, multi-touch interaction around small devices [11]; capacitive sensing techniques enabling the detection of touch events on humans, screens, liquids, and everyday objects [12]; electromyography systems that measure muscle tension [13,14,15]; and even acoustic sensing systems [16,17,18,19]. The latter range from commercial miniature ultrasonic sensors on chips to recent developments in ultrasonic gesture sensing methods through on-body acoustics. Particularly effective are methods where the acoustic signals of various frequency ranges are induced and the response analysed for interactions with the anatomy of our body. Previous research showed that this can be used for sensing both on-object [16,17,18] as well as mid-air gestures [19].

Despite numerous advances in gesture interaction systems, detecting gestures on-objects remains challenging, particularly if the object is not instrumented, as previously explained. However, recent advances in radio-frequency sensing technologies, especially gesture recognition with miniaturised mm-wave radar sensors, offer a new alternative to on-object interaction systems. We discuss these in the following sections.

### 2.2. RF Sensing Technologies

Regardless of popular technologies used for implementing gesture recognisers such as RGB [20,21,22,23] or infrared (IR) [24,25,26,27,28] cameras, Radio-frequency solutions including radar [29,30], Wi-Fi [31,32,33], GSM [34], and RFID [35] offer several advantages. Above all, RF sensing technologies are insensitive to light, which usually affects the camera and especially, IR-based solutions. RF sensing also does not require an elaborate setup of various sensors on or around users. In addition, the RF signal can penetrate non-metallic surfaces and can sense objects and their movements through them.

RF sensing has been used for analysing walking patterns or gait [36,37,38], tracking sleep quality and breathing patterns [39,40], and recognising movements of body parts such as hands for interactive purposes [31,35,41,42,43,44,45]. The radars used in these studies operated at various frequencies, ranging from 2.4 GHz [40,42] to 24 GHz [39,43].

### 2.3. Millimetre-Wave Radar-On-Chip Sensors

To detect and recognise fine-grained interactions, it is necessary to increase the radar’s spatial resolution. Recently, radar chips working at frequencies ranging from 50 to 70 GHz, have been introduced and studied [30,46]. Since these sensors operate in the millimetre range, they allow for the tighter integration of the circuit due to the reduced size of different passive (non-moving) components and low-power requirements [30]. These properties also enable inexpensive and large-scale manufacturing.

More importantly, because of the increased spatial resolution, such chips are very effective in detecting close-proximity, subtle, nonrigid motions mostly articulated with hands and fingers (i.e., rubbing, pinching, or swiping) [47,48] or with small objects (i.e., pens) [46] as well as large gestures in 3D space [10]. This opens up a plethora of possibilities for precise close-range micro gesture interactions in a variety of applications, including wearable, mobile, and ubiquitous computing.

Recent research has explored mm-wave radar sensing for interaction with everyday objects and in augmented reality scenarios [1,49], as well as in creating music [50,51], or for distinguishing various materials when placed on top of it [45,52]. It should be mentioned that there are two standard approaches for gesture recognition with mm-wave radars: one feeds the raw signals or derived images (i.e., Doppler images) directly into the classifier [47,53] enabled by for example the Google Soli sensor, while the other approach applies different beamforming vectors to extract/track the location before feeding it to a classifier [10,54], which can be done with other mm-wave sensors such as the IWR1443 board from Texas Instruments. What is missing in the research literature, however, is an investigation of gesture recognition performance as gestures are executed on various objects, which is the focus of our work.

## 3. Materials and Methods

We describe the three model architectures considered for on-object gesture detection with the radar system. The first one is a hybrid architecture combining a convolutional (CNN) and a long short-term memory (LSTM) neural network (hereafter referred to as the hybrid model) that has been used in previous work on radar sensing of mid-air gestures [48]. We then propose two alternative model architectures: a spatio-temporal 3D CNN architecture (referred as the Conv3D model), and a 2D CNN architecture where temporal information is implicitly encoded by a spectrogram image (referred as the spectrogram model). Since the hybrid CNN+LSTM architecture was previously used for mid-air detection [48], we ran the evaluation of mid-air detection for all three model architectures. This was conducted in order to understand how our two novel architectures perform compared to the baseline hybrid model, for which we use the data set and results provided by Wang et al. [48].

The following subsections focus on the on-object gesture detection research starting with the description of on-object gesture selection process. Then, we depict the system for recording the on-object gestures including the explanation of the object used in the study. This is followed by the subsection on the data collection. Finally, we describe all the experiments we performed on on-object gesture detection.

### 3.1. Model Architectures

#### 3.1.1. Hybrid CNN+LSTM Model

**Figure 2:**
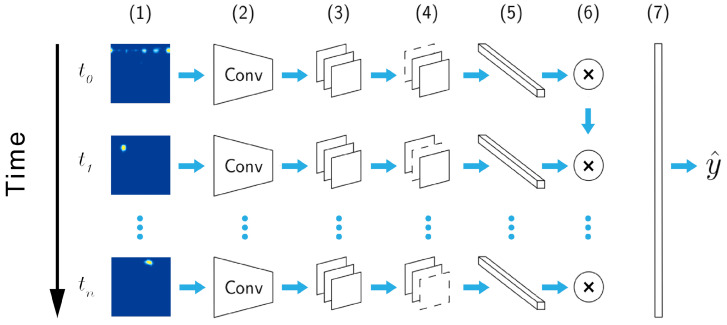
Hybrid deep learning model architecture. Range Doppler images (1) are processed with a stack of CNN layers (2) that extract feature maps followed by maxpooling (3) and spatial dropout (4) layers. Then, a fully connected layer (5) creates the feature vectors for a recurrent LSTM layer with a dropout (6) and finally, a softmax layer (7) outputs the gesture class prediction ($\hat{y}$).

The hybrid model, depicted in Figure 2, is a deep CNN+LSTM architecture inspired by previous work [47,48,53,55,56,57]. Such architecture has also been used successfully for the radar sensing of mid-air gestures [48,53], and is thus considered as the baseline model architecture.

In the hybrid model, each frame (Doppler image) is processed by a stack of 32×64×128 CNN layers with 3×3 filters to capture spatial information. The resulting frame sequence is further processed in a recurrent fashion by the means of an LSTM layer (embedding size of 128) to capture temporal information, and eventually classified with a softmax layer. The model has 2.4 M weights, which is rather small for today’s standards. Each convolutional layer automatically extracts feature maps from input frames that are further processed by maxpooling and spatial dropout layers. The maxpooling layers (pool size of 2) downsample the feature maps by taking the largest value of the map patches, resulting in a local translation invariance.

Crucially, the spatial dropout layer (drop rate of 0.25) removes entire feature maps at random, instead of individual neurons (as it happens in regular dropout layers), which promotes independence between feature maps, and consequently improves performance. The LSTM layer uses both a dropout rate and a recurrent dropout rate of 0.25. The softmax layer has dimensionality of 5 or 11, since we experiment with 5 and 11 gestures in this paper.

#### 3.1.2. Conv3D Model

**Figure 3:**
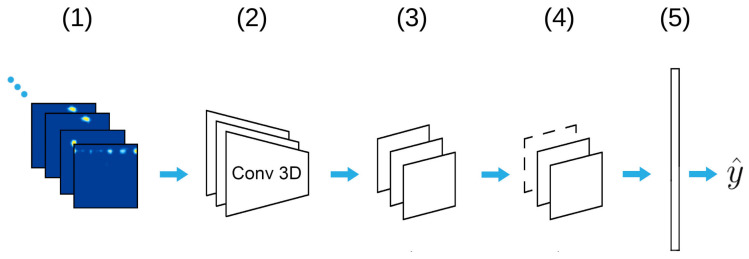
Conv3D deep learning model architecture. Range Doppler images (1) are processed with a stack of Conv3D layers (2) with kernel size 2×2×2 that extracts feature maps followed by 3D maxpooling (3) and spatial dropout (4) layers. Layers (2)–(4) are stacked in blocks of 32, 64, 128, and 256 units. Then, a fully connected layer creates feature vectors, which are fed into the softmax layer (5) for class prediction ($\hat{y}$).

Previous work shows that the spatio-temporal 3D CNN (Conv3D) architecture is an effective tool for the accurate action recognition of image sequences [58,59]. Since Soli provides a sequence of Doppler images through time, we developed a custom Conv3D architecture (Figure 3). In the Conv3D model, the range Doppler images are processed with a stack of Conv3D layers that extracts feature maps followed by 3D maxpooling and spatial dropout layers. Then, a fully connected layer creates feature vectors, which are fed into the softmax layer for classification. The model has 3.5 M weights, which is considered rather small for today’s standards. For further details, we refer the reader to Appendix A.

#### 3.1.3. Spectrogram CNN Model

**Figure 4:**
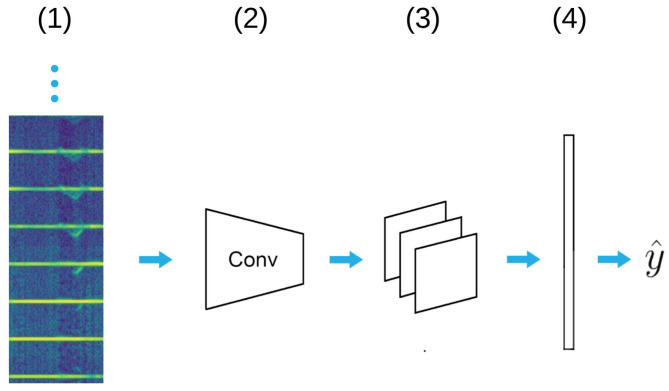
A spectrogram image (1) is processed with a stack of 32×64×128×256 convolutional layers with 3×3 kernel to capture spatial information. Each convolutional layer extracts feature maps from the input image and is further processed by maxpooling layer (3). Finally, a softmax layer outputs the gesture class prediction ($\hat{y}$).

**Figure 5:**
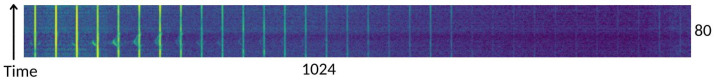
A spectrogram image pre-processed with the dB range [−16, 0]. The image is generated by flattening a sequence of 80 range Doppler images. Each row represents one range Doppler image of 32×32=1024 px.

This model (Figure 4) is a standard 2D CNN architecture where both the temporal and spatial information are encoded by a spectrogram image. Here, a spectrogram stores information about one gesture instance, i.e., a sequence of 80 32×32 range Doppler images. Range Doppler images were generated and pre-processed following the procedures explained in Section 3.4 and were flattened into 1024 bins and stacked on top of each other, resulting in a spectrogram image of 1024 × 80 pixels (Figure 5).

Each spectrogram image is processed by a stack of 32×64×128×256 convolutional layers with 3×3 kernels. Each CNN layer extracts feature maps from the input images and is further processed by the maxpooling layer (pool size of 2) and finally classified with a softmax layer. The model has 0.9 M weights, which is the smallest of the three models considered. This is particularly desirable for applications in emended systems. For further details, we refer the reader to Appendix B.

#### 3.1.4. Evaluating Model Architectures on Mid-Air Gestures

The goal of this evaluation was two-fold. First, to ensure that our implemented baseline model architecture (i.e., hybrid model) was of comparable performance to previously reported results [48]. Second, to compare it with the two alternative model architectures. We ran the evaluation on 11 mid-air gestures from the publicly available data set [48] which holds 2750 labelled instances (10 subjects × 11 gestures × 25 repetitions per gesture). For all the details about data collection and pre-processing, we refer the reader to the original paper [48]. The model training and evaluation was run as described in Section 3.5.

The results in Table 1 show that the accuracy of the hybrid model performs, similarly to the one reported by Wang et al. [48] (90.6% to 87.17%, respectively). However, it is important to note that the evaluation is not conducted in the exact same way, as we did not follow the same cross-validation procedure in our experimentation. Irrespective of this shortcoming, it is very unlikely that such cross-validation would drastically change the outcome. We can conclude that our replicated model is at least on equal footing to the one reported by Wang et al. [48]. Table 1 also shows that the proposed alternative models clearly outperform the current state-of-the-art hybrid model with an accuracy gain of 8%, achieving an almost perfect accuracy of above 98%.

### 3.2. Gesture Set

**Figure 6:**
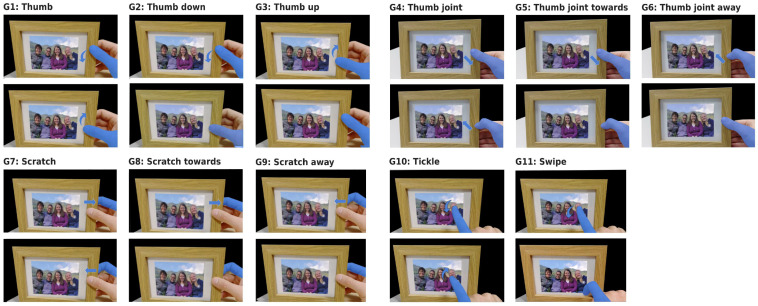
Gesture set for on-object interaction based on the existing literature and heuristic analysis. Gestures are divided in (i) bidirectional gestures—gestures which include movements in two directions (G1, G4, G7, G10); and (ii) unidirectional gestures—gestures which include movement in one direction only (G2 and G3, G5 and G6, G8 and G9, G11).

From this subsection forward, we solely focus on the main aim of this research—the on-object gesture recognition. The selection of 11 on-object gestures (see Figure 6) was based on the review of successful radar-based micro-gesture detection systems [47,48] as well as on the heuristic analysis considering the capabilities of radar sensing systems. In this analysis, special attention was given to gesture ergonomics—the authors experimented with and executed various gestures and only selected the ones they could execute with ease. The chosen set of 11 gestures substantially overlaps with the recently published elicitation study on grasping micro-gestures [60]. For example, our Thumb gestures (G2 and G3) are mapped to actions increase/decrease, whereas our Thumb joint gestures (G5 and G6) are mapped to actions next/previous, and our scratch gesture (G9) is mapped to reject/delete action.

The 11 gestures can be divided into two groups: (i) bidirectional gestures—gestures which include movements in two directions (e.g., thumb and scratch gestures); and (ii) unidirectional gestures—gestures which include movement in one direction only (e.g., thumb up and thumb down gestures). From a radar sensing perspective, the bidirectional gestures are more difficult to detect since the radar sensors have difficulties inferring the direction of movements and the movements in these gestures are the key identifier of the gesture (e.g., the main difference of thumb up and thumb down gesture is only the direction of the movement). This is especially the case if range Doppler images are used: these images only show information about the range and velocity of a moving target and do not include information about the direction of movement if it happens outside of the range axis.

To reduce the difficulty of gesture detection problem, we ran several experiments on a reduced gesture set (G1, G4, G7, G10 and G11) where failing to correctly identify the direction of movement would not affect recognition performance.

### 3.3. Sensing System

**Figure 7:**
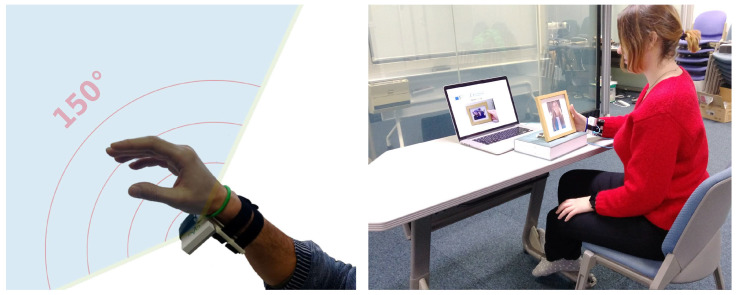
(**Left**): We used a wrist-mounted Google Soli for sensing micro-gestures on physical objects; (**Right**): In our recording session, participants sat at a desk while interacting with a standing wooden photo frame.

We used the same sensing system as in previous work [1]. The Google Soli sensor was mounted on a wrist using a 3D-printed wristband (see Figure 7 left). The sensor was positioned to illuminate the fingers, maximising the amount of captured reflected signal caused by the hand and finger movements. The sensor was connected to a laptop computer via a USB cable. The form factor of our prototype implementation is large because of the original Soli sensor used; however, the radar-on-chip technology has already been miniaturised to the level where it is integrated into smartphones (Google Pixel 4). Therefore, it is very likely that these chips will soon become available on wearables such as smart watches and smart bands, and could be positioned in a similar way as in our experiment.

### 3.4. Data Collection and Pre-Pocessing

We recorded 8.8k labelled instances (11 gestures, 10 users, 20 gesture repetitions, 3–5 sessions) following the same procedure as in our previous work [1]. The gestures were recorded whilst being executed on a standing wooden photo frame. We selected a photo frame as our exemplar object since such frames are present in nearly every home as well as office, and they offer numerous options for object augmentation [61]. Furthermore, our frame is made of non-conductive materials (wood, plastic, glass, and cardboard) and should therefore be at least partially transparent to the radar signal.

Ten participants (6 male, 4 female, aged 23–50) were sitting at a desk whilst executing the gestures on a photo frame as in Figure 7 right. All instructions were provided on a laptop in front of them. An animated image of the gesture to be executed next with its name was shown before participants started recording each next gesture. After a beep sound was played, participants had to execute the gesture. This was repeated 20 times for each gesture. The order of gestures was randomised and after each round, the sensor was reset (the clutter map of the radar sensor was rebuilt). Participants were also asked to repeat the gesture if the one they executed did not match the gesture shown on the image (e.g., the user executed the scratch gesture instead of the thumb gesture).

The Soli sensor was configured to record on 4 channels at 1000 Hz with the adaptive clutter filter disabled. The recorded files were pre-processed at varying range settings, generating 4 range Doppler images for each frame. Since this process is slow due to the vast amount of images created, we experimented with only 5 dB range settings covering the whole sensor range (i.e., [−2, 0], [−4, 0], [−8, 0], [−16, 0] and [−32, 0]). Because Soli computes range Doppler images for each receiver antenna, we averaged them to ensure a robust frame representation. Furthermore, images were greyscaled and sequences were resampled to 100 Hz. As a reference, each recorded gesture took 0.8 s or 80 timesteps.

### 3.5. Model Training and Evaluation

We created random splits dedicating 50% of the data to model training, 20% to model validation, and the remaining 30% to model testing. The test data are held out as a separate partition, which simulates unseen data. The models were trained in batches of 32 sequences using categorical cross-entropy as a loss function. We used the Adam optimiser with the learning rate of η=0.001 and exponential decay rates of $\beta_{1}=0.9,\beta_{2}=0.999$. The maximum number of epochs was set to 200, but we also set an early stopping criteria of 50 epochs. That is, the training stopped if the validation loss did not improve after 50 consecutive epochs, and the best model weights were retained.

We ran several experiments (explained within the subsections below) to uncover the relationships between dB range setting and gesture detection performance. All experiments were repeated three times with different random seeds to minimise the bias of data split.

#### 3.5.1. Effect of dB Range Setting on Model Performance

To analyse the effect of the dB range setting on gesture detection performance, we evaluated 15 different scenarios varying the model architecture (i.e., hybrid, Conv3D, spectrogram) and the dB range settings ([−2, 0], [−4, 0], …, [−32, 0]). To reduce the inherent difficulty of the gesture classification problem, we ran the experiment on a reduced gesture set including G1, G4, G7, G10 and G11 (for the rationale on the gesture selection, we refer the reader to Section 3.2).

#### 3.5.2. Relationship between User and dB Range Setting

To uncover whether the dB range was a user-dependent design parameter, we evaluated 70 different scenarios varying our two proposed model architectures (i.e., Conv3D and spectrogram), 5 dB range settings ([−2, 0], [−4, 0], …, [−32, 0]), and 7 data partitions (each based on a different user). We only have 7 partitions because we removed 3 users from this evaluation (users 2, 4 and 10), since they did not participate in all 5 recording sessions, which resulted in partition sizes which were too small for testing. Again, we ran the experiment on a reduced gesture set including G1, G4, G7, G10 and G11 (for the rationale on the gesture selection, we refer the reader to Section 3.2).

#### 3.5.3. Evaluation of Calibrated System

To evaluate the performance of the calibrated system, we evaluated 3 scenarios where the only parameter we varied was the model architecture (i.e., hybrid, Conv3D and spectrogram). We used the optimal dB range setting we identified in Section 4.1, which is [−16,0]. This time, we trained and evaluated our classifiers on the full on-object gesture set, making the gesture detection task much harder. For each scenario, we again trained and evaluated 3 classifiers and reported the averaged results.

## 4. Results

### 4.1. Effect of dB Range Setting on Model Performance

**Figure 8:**
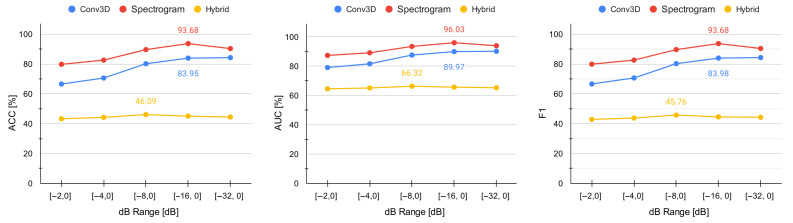
Relationship between accuracy and dB range settings. There is a significant impact of the dB range settings on the performance as long as the model performs reasonably well overall. The optimum for both alternative architectures (Conv3D and spectrogram) is at [−16, 0].

The results in Table 2 show the poor performance of the hybrid architecture for the radar-based on-object gesture detection. The performance did not significantly improve through the whole dB range (accuracy remained between 43% and 46%). This clearly indicates that alternative model architectures are needed. The results in Table 2 and Figure 8 also reveal that there is a strong effect of the dB range setting on the recognition performance for our Conv3D and spectrogram architectures, since the accuracy improved by 20 and 14 percentage points, respectively.

Furthermore, the results also showed that (1) underestimating the dB range is worse than overestimating it; and (2) our two proposed model architectures have an optimum dB range at [−16, 0]. These new facts can result in making a more informed decision when calibrating the sensor.

### 4.2. Relationship between Users and dB Range Settings

**Figure 9:**
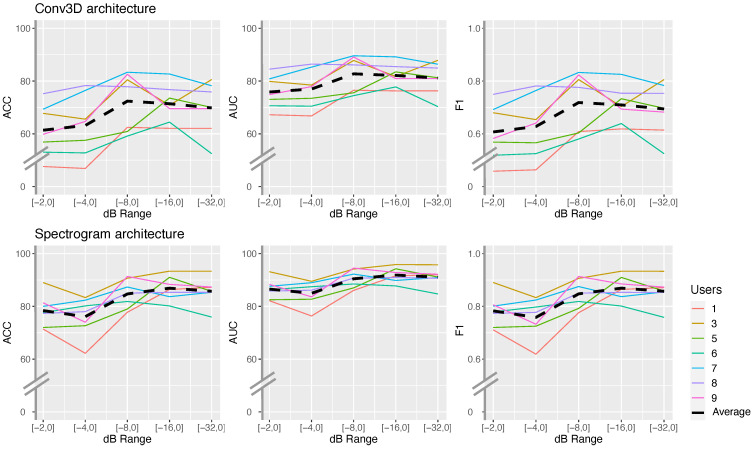
Per user evaluation of model architectures at varying dB range settings. The graphs show a similar trend across all users (e.g., drastic improvements stop at dB range [−8,0]).

The results in Figure 9 show that users do not have a strong effect on calibrating the sensor, since the same trend can be observed for all users. For example, there is a clear cutoff at the dB range [−8,0] after which only marginal improvements were observed. This allows us to conclude that the dB range sensor calibration is user-independent.

### 4.3. Evaluation of Calibrated System

The results in Table 3 show that our Conv3D and spectrogram architectures clearly outperform the hybrid architecture. This is the case for both the reduced (five gestures) and full gesture sets (11 gestures). In addition, after more than doubling the number of gestures in the full set, recognition performance remains high for our proposed architectures: accuracy is 78.89% for Conv3D and 74.55% for the spectrogram model, respectively. However, overall this performance falls short when it comes to deploying a usable gesture detection system.

The results also show that there is no clear best architecture candidate, as the Conv3D model outperformed the spectrogram-based CNN for the full gesture set (accuracy of 78.89% vs. 74.55%), whilst the opposite was observed for the reduced gesture set (accuracy 83.95% vs. 93.65%). This can be explained by the fact that gestures in the reduced set are not so dependent on the directionality of the movement; therefore, any minimally encoded temporal information does suffice. On the contrary, gestures in the full set are more dependent on the movement directionality and are thus more difficult to recognise. Therefore, a more sophisticated model architecture is necessary.

**Figure 10:**
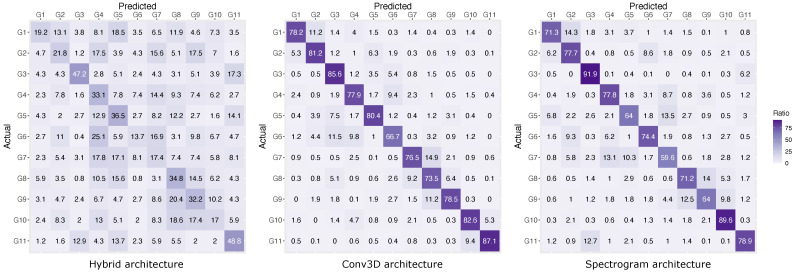
Confusion matrix for the full gesture set (11 gestures).

**Figure 11:**
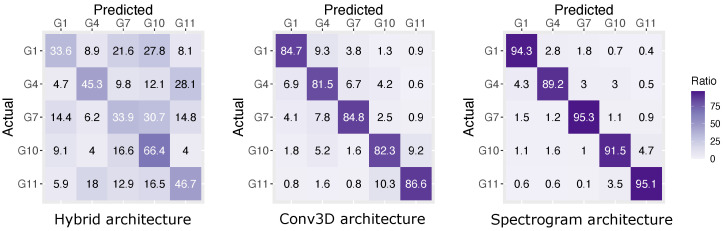
Confusion matrix for the reduced gesture set (5 gestures).

Looking at the confusion matrices in Figure 10 and Figure 11, we can make several interesting observations. For example, all model architectures have difficulties distinguishing between bidirectional gesture pairs, where the main distinction between the two is the direction of the movement. This is, for example, the case for ‘scratch towards’ and ‘scratch Away’ gestures (G8 and G9). Furthermore, the confusion matrix for the full gesture set in Figure 10 also reveals that gestures ‘thumb’, ‘thumb joint’ and ‘scratch’ (i.e., G1, G4 and G7) perform substantially worse than gestures ‘tickle’ and ‘swipe’ (i.e., G10 and G11). This was not observed in the reduced gesture set (Figure 11).

## 5. Discussion

This section was structured following the three research questions which we set out to answer: Can radar sensing be used for robust on-object gesture detection? How does dB range affect gesture detection performance and is this user dependent? What is the potential gain of calibrating the dB range correctly and how can on do it? These are followed by discussing our results beyond on-object gesture detection and concluding with the limitations and future work sections.

### 5.1. Robust On-Object Gesture Detection Using Radar Sensing

To the best of our knowledge, our previous work is the only research on on-object gesture detection using a mm-wave radar sensor. In previous work, we concluded that on-object gesture detection was not possible with traditional machine learning models, as the maximum classification accuracy on a four-gesture set (i.e., G1, G7, G10 and G11) was only 55%. However, this result was obtained with random forest and support-vector machine classifiers which were fed with core features provided by the Google Soli SDK [1]. Hence, we hypothesised that a substantial improvement should be possible if the detection pipeline included other sensor features, such as range Doppler images, and more advanced machine learning methods, such as convolutional and/or recurrent neural networks.

Our results in Section 4.3 initially failed to confirm this hypothesis. The state-of-the-art hybrid architecture, which was successfully used in several mid-air gesture detection scenarios [48,53], failed to improve the performance of on-object gesture detection. On a five-gesture set (i.e., G1, G3, G7, G10 and G11), we observed an accuracy of only 45.08%. We hypothesise that the reason behind this low accuracy is the sensitivity of the hybrid model to noise in the input signal caused by the occluding object on which gestures are being executed.

Our two alternative model architectures for radar sensing achieved a significant improvement in recognition accuracy. On the reduced set of five gestures, accuracy improved from 44.08% for the hybrid model to 83.95% and 93.68% for Conv3D and spectrogram architecture, respectively. Moreover, this improvement gain increased even further on the full gesture set of 11 gestures, from 29.23% for the hybrid architecture to 78.89% and 74.55% for Conv3D and spectrogram architectures, respectively.

A detailed analysis of the confusion matrix revealed that our models have problems with distinguishing between bidirectional gesture pairs, where the main distinction between the two is the direction of movement (such as ‘scratch towards’ and ‘scratch Away’ gestures). This is likely the case because range Doppler images only hold information about the range and velocity of the moving target, making it difficult to infer the direction of movement, which does not occur along the range axis (i.e., moving closer or further away from the sensor).

Furthermore, the confusion matrix for the full gesture set (Figure 10) revealed that the ‘thumb’, ‘thumb joint’, and ‘scratch’ gestures (G1, G4 and G7) performed substantially worse than ‘tickle’ and ‘swipe’ gestures (G10 and G11). This is likely the case because these gestures also include bidirectional variations (e.g., ‘thumb up’, ‘thumb down’, ‘scratch towards’, ‘scratch away’, etc.). These bidirectional variations have several similar movement characteristics, which makes classification challenging. Therefore, these should be avoided when deciding on the gesture set.

### 5.2. Selecting Optimal dB Range Setting

As hypothesised, there is a strong effect of the dB range setting on recognition performance since, over the full dB range, the accuracy of Conv3D and spectrogram models improved by 20 and 14 percentage points, respectively, which is an impressive gain. However, this was not observed for the hybrid model, which performed poorly across the whole dB range. Therefore, selecting the optimal dB range setting may only improve accuracy for models that already perform reasonably well.

Perhaps surprising is the fact that overestimating the dB range setting is preferable to underestimating it. Moreover, the degradation in recognition accuracy is only marginal when close to the end of the dB range. This indicates that adding potential noise to the signal (by increasing the dB range) is much more beneficial than missing out on potentially relevant information (by decreasing the dB range). Therefore, for on-object gesture detection scenarios, one should stick to the maximal dB range ([−32, 0]) if no time can be spared on fine-tuning the detection pipeline. This is also how the Soli SDK configures the sensor by default. A much smaller dB range when performing mid-air gestures was so far used in the literature ([−2, 0] [53]). However, there was no justification as for why this setting was selected. Therefore, until now, it was unclear what the optimal dB range setting is for mid-air gesture detection and if the same guidance would apply to on-object gesture detection.

The results also suggest that the optimal dB range is at [−16, 0] for both alternative architectures (Conv3D and spectrogram) and that such a range setting is user-independent. This is an important finding because it offers information for optimising sensor calibration methods, for the three following reasons. First, personalised calibration is not required, so the sensor needs to be calibrated only once for each sensing scenario. Second, since the calibration process is user-independent, it does not really matter which users are selected for this process. Third, one does not need to calibrate the system on the whole data set, but can use a smaller data partition. The latter reason also bears its own importance, as a grid search strategy for the optimal dB range requires the extensive generation of images (i.e., four images are generated per frame for each dB range settings), as well as training and evaluation for numerous models. These processes are inherently resource-hungry, thus further optimisations are needed.

### 5.3. Beyond Gestures On-Objects

We also compared the three model architectures on a publicly available mid-air gesture set, and found that our two proposed model architectures (Conv3D and spectrogram) clearly outperform the current state-of-the-art architecture (hybrid) with a significant accuracy gain, achieving almost perfect recognition performance (98.6% and 98.53%, respectively). Particularly interesting is the lightweight spectrogram model that is requiring three times fewer weights (0.9 M weights) than the hybrid model. This makes it very suitable for embedded applications where resources, such as computational power and battery, are at a premium.

### 5.4. Limitations and Future Work

We explored the possibility of detecting micro-gestures on physical objects. However, we based our findings on experimentation with a single object (i.e., a standing wooden photo frame). Will our findings generalise to other objects? We hypothesise this is the case as long as the object is radar-friendly (built with materials that are transparent to the radar signal). The question is how transparent are various materials within the operational frequency range of a mm-wave radar sensor? Even though a few tabulations of material properties are available in the literature [62,63], to the best of our knowledge, none exist for a large variety of everyday materials we can find in our homes, offices and other environments. Future research should provide such tabulation values and explore how they could be applied to the on-object gesture detection scenarios, enabling more informed choices of object selection and perhaps, through distortion modelling, enhance system performance.

The performance comparison of the two alternative model architectures (i.e., Conv3D and spectrogram) against standard machine learning methods (Table 4) shows that the alternatives perform significantly better. However, it is important to note that they do not use the same input data. The performance of alternative models could be further improved by acquiring more data for training and evaluation, since it will likely lead to higher resilience to noise. Furthermore, the range Doppler signal could be combined with other outputs from the sensor’s signal processing pipeline. This would work as long as such additional signals may introduce new information to the gesture detection process.

As discussed in Section 2.1 and Section 2.2, alternative sensing modalities exist for on-object gesture detection. However, a direct performance comparison of these with our proposed radar-based gesture detection method is not possible, as there are many differences between the evaluation procedures. For example, the gesture sets are hardly comparable because they differ in gesture type (e.g., static vs. dynamic gestures), amount of movement (e.g., moving a finger, wrist, hand, or arm), or type of movement (e.g., single-direction, multiple-directions). Such a comparison would be a valuable addition to the body of knowledge and should be conducted in future work with comparable evaluation procedures.

Finally, the radar sensing approach we used in this work is based on range Doppler images but there are also other approaches to radar sensing, such as the use of beamforming vectors, which can track the location of reflections [10,54]. Perhaps such a radar sensing method would perform better when classifying bidirectional gestures. Therefore, on-object gesture detection should also be explored with this alternative radar sensing method.

## 6. Conclusions

We focused on micro-gesture detection on physical objects, aimed at solving the missing interface problem. We first designed a set of gestures based on previous research on radar-based gesture detection and grasping micro-gestures [47,48,60]. Then, we recorded 8.8k of labelled instances of these gestures on a standing wooden photo frame and developed two alternative model architectures Conv3D and spectrogram-based CNN models. We conducted several experiments to evaluate and compare these novel architectures with the baseline hybrid model architecture, as well as to explore the role of sensor calibration.

This paper is the first to show that a robust gesture detection on objects is possible with radar sensing as long as: (i) the gesture set is carefully chosen to include radar-friendly gestures (e.g., avoiding gestures where the direction of movement is the main signifier); (ii) the chosen object is radar-friendly (i.e., it has low opacity for the radar signal); (iii) the dB range of the sensor is properly calibrated; and (iv) the detection pipeline includes advanced sensor features (range Doppler images) and one of our proposed alternative architectures (Conv3D or spectrogram).

We also uncovered the relationship between the dB range setting and detection performance, highlighting key design guidelines for sensor calibration: (i) underestimating the dB range is worse than overestimating it, thus one should set the sensor to the maximum dB range setting when in doubt; and (ii) dB range calibration is user independent and it thus only needs to be done once and on any combination of users.

## Figures and Tables

**Table 1:** Evaluation of three model architectures for mid-air gesture detection. The classifiers were trained to recognise 11 mid-air gestures from publicly available gesture set [48].

Model Architecture	Num of Gestures	Num of Weights	ACC	AUC	Precision	Recall	F1
Hybrid	11	2,825,515	90.56	94.81	90.64	90.56	90.50
Conv3D	11	3,499,243	98.60	99.23	98.65	98.60	98.61
Spectrogram	11	912,203	98.53	99.19	98.56	98.53	98.53

**Table 2:** Performance of different model architectures at varying dB range settings.

Model Arhitecture	dB Range	Num of Weights	ACC	AUC	Precision	Recall	F1
Hybrid	[−2,0]	2,825,515	43.28	64.56	43.04	43.28	42.89
Hybrid	[−4,0]	2,825,515	44.22	65.14	43.94	44.22	43.80
Hybrid	[−8,0]	2,825,515	46.09	66.32	46.18	46.09	45.76
Hybrid	[−16, 0]	2,825,515	45.08	65.69	45.89	45.08	44.56
Hybrid	[−32, 0]	2,825,515	44.45	65.28	44.48	44.45	44.29
Conv3D	[−2,0]	3,499,243	66.56	79.10	67.36	66.56	66.64
Conv3D	[−4,0]	3,499,243	70.61	81.63	71.10	70.61	70.65
Conv3D	[−8,0]	3,499,243	80.19	87.62	80.72	80.19	80.22
Conv3D	[−16, 0]	3,499,243	83.95	89.97	84.23	83.95	83.98
Conv3D	[−32, 0]	3,499,243	84.27	90.17	84.61	84.27	84.32
Spectrogram	[−2,0]	912,203	79.81	87.36	80.91	79.81	79.92
Spectrogram	[−4,0]	912,203	82.60	89.13	83.07	82.60	82.62
Spectrogram	[−8,0]	912,203	89.65	93.50	89.82	89.65	89.65
Spectrogram	[−16, 0]	912,203	93.68	96.03	93.82	93.68	93.68
Spectrogram	[−32, 0]	912,203	90.35	93.98	90.82	90.35	90.41

**Table 3:** Evaluating model architectures at optimal dB range [−16,0].

Model	Num of Gestures	Num of Weights	ACC	AUC	Precision	Recall	F1
Hybrid	11	2,825,515	29.33	61.07	30.36	29.23	28.94
Conv3D	11	3,499,243	78.89	88.39	79.43	78.89	78.95
Spectrogram	11	912,203	74.55	86.00	75.11	74.55	74.34
Hybrid	5	2,825,515	45.08	65.69	45.89	45.08	44.56
Conv3D	5	3,499,243	83.95	89.97	84.23	83.95	83.98
Spectrogram	5	912,203	93.68	96.03	93.82	93.68	93.68

**Table 4:** Comparison of proposed model architectures with standard machine learning techniques on the same gesture set.

Model	Num of Gestures	Input Data	ACC
Hybrid	5	Range Doppler Images	45.08
Conv3D	5	Range Doppler Images	83.95
Spectrogram	5	Range Doppler Images	93.68
Random Forest [1]	4	Soli Core Features	55.00
Support-vector Machine [1]	4	Soli Core Features	50.00

**Figure A1:**
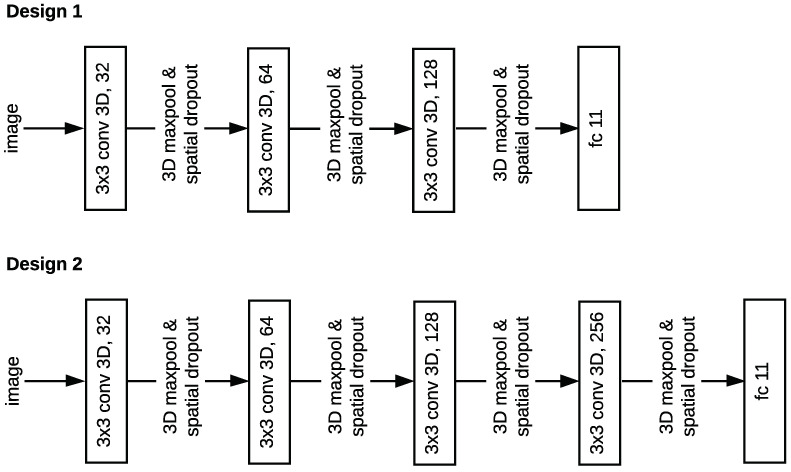
The evolution of Conv3D model architecture from Design 1 to final Design 2.

**Figure A2:**
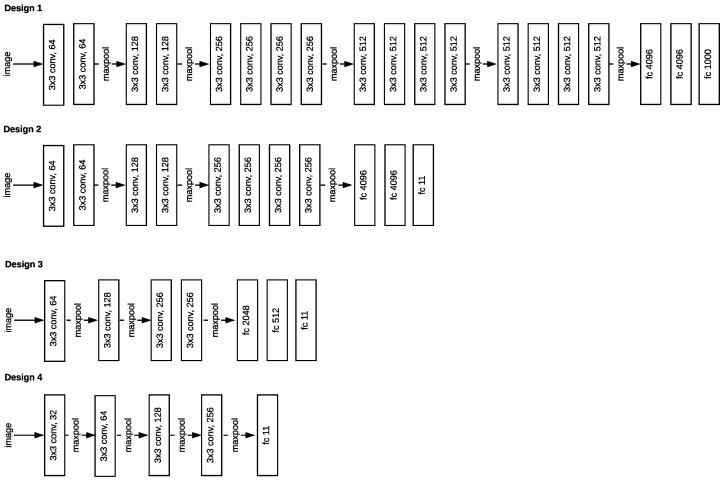
The evolution of spectrogram model architecture from VGG-19 architecture (Design 1) to the final model architecture (Design 4).

## Data Availability

We used two public data sets: (1) a set of mid-air gestures that can be found at https://github.com/simonwsw/deep-soli (accessed on 28 April 2020); and (2) our set of on-object micro-gestures that we make available at https://gitlab.com/hicuplab/mi-soli (accessed on 21 August 2021).

## Appendix A. Designing the Conv3D Architecture

Previous work showed that the spatio-temporal 3D CNN (Conv3D) architecture is an effective tool for the accurate action recognition of image sequences [58,59]. As the 3D convolutional layers already hold temporal information about gestures, there is no need to use LSTM layers, as shown in Design 1 in Figure A1. Training and evaluating Design 1 revealed that this model was 80% accurate on the reduced gesture set. We decided to increase the number of CNN layers, as shown in Design 2 in Figure A1. Training and evaluating Design 2 on the same reduced gesture increased accuracy to 84%.

## Appendix B. Designing the Spectrogram CNN Architecture

Our spectrogram mode is a convolutional neural network (CNN) based on the VGG-19 architecture [64,65]. However, this architecture was substantially modified. First, we removed all convolutional layers having 512 frames, reducing the number of layers by 8 (see Design 1 and 2 in Figure A2). We did this in order to accommodate the model for our input image size. Still, this model has a large number of trainable weights (530 M). Training and evaluation revealed poor performance, with 9.26% accuracy on the full gesture set. We hypothesised that the high number of weights caused overfitting, due to a relatively small data set of 8.8 k labelled instances (approximately 800 instances per gesture class). Therefore, we reduced the number of weights by reducing the number of convolutional layers by half, as well as the number of neurons in the fully connected layers (see Design 3 in Figure A2). These architecture modifications reduced the model size to 2.9 M weights and improved the classification accuracy on reduced the gesture set to 85%.

To improve our architecture even further, we decided to add a small convolutional layer at the beginning of our architecture (as is the case of the hybrid architecture) and removed fully connected layers all together (see Design 4 in Figure A2). This reduced the size of our model even further to 0.9 M weights and resulted in an accuracy improvement from 85% to 93% on the reduced gesture set.
